# Intraocular Pressure Changes during Accommodation in Progressing Myopes, Stable Myopes and Emmetropes

**DOI:** 10.1371/journal.pone.0141839

**Published:** 2015-10-30

**Authors:** Yan Liu, Huibin Lv, Xiaodan Jiang, Xiaodan Hu, Mingzhou Zhang, Xuemin Li

**Affiliations:** Department of Ophthalmology, Peking University Third Hospital, Beijing, China; National Eye Institute, UNITED STATES

## Abstract

**Purpose:**

To investigate the changes of intraocular pressure (IOP) induced by 3-diopter (3 D) accommodation in progressing myopes, stable myopes and emmetropes.

**Design:**

Cross-sectional study.

**Participants:**

318 subjects including 270 myopes and 48 emmetropes.

**Methods:**

195 progressing myopes, 75 stable myopes and 48 emmetropes participated in this study. All subjects had their IOP measured using iCare rebound tonometer while accommodative stimuli of 0 D and 3 D were presented.

**Main Outcome Measures:**

IOP values without accommodation and with 3 D accommodation were measured in all subjects. Baseline IOPs and IOP changes were compared within and between groups.

**Results:**

There was no significant difference in IOPs between progressing myopes, stable myopes and emmetropes when no accommodation was induced (17.47±3.46, 16.62±2.98 and 16.80±3.62 respectively, p>0.05). IOP experienced an insignificantly slight decrease after 3 D accommodation in three groups (mean change -0.19±2.16, -0.03±1.68 and -0.39±2.65 respectively, p>0.05). Subgroup analysis showed in progressing myopic group, IOP of children (<18 years old) declined with accommodation while IOP of adults (≥18 years) increased, and the difference was statistically significant (p = 0.008). However, after excluding the age factor, accommodation induced IOP changes of high progressing myopes (≤-6 D), low, moderate and non-myopes (>-6 D) was not significantly different after Bonferroni correction (p = 0.838).

**Conclusions:**

Although no difference was detected between the baseline IOPs and accommodation induced IOP changes in progressing myopes, stable myopes and emmetropes, this study found accommodation could cause transient IOP elevation in adult progressing myopes.

## Introduction

Biomechanics has long been considered to play a role in the pathogenesis of myopia[1–3]. Intraocular pressure (IOP), as a kind of mechanical force, has been hypothesized to be one of several factors implicated in the initiation and development of myopia[4–7]. Some studies[4, 6] reported that the mean IOP value of myopes was significantly higher than that of emmetropes. Accordingly, some authors proposed that elevated IOP could result in myopic axial elongation and promote myopia development. However, there were few longitudinal studies[8]that could elucidate whether the IOP elevation was the cause of axial elongation or just a matter of result. Moreover, there were several studies[5, 7] reported conflicting results that no difference of IOP was identified between myopes and emmetropes. Until now, the exact influence of IOP on axial length and human refractive error development remains poorly understood.

Our recent study[9] found that instead of baseline IOP values, it was the IOP variations that might participate in myopia progression. We found that baseline IOP levels were not significantly different, but the IOP experienced a transient elevation in progressing myopes and decreased a little in emmetropes when accommodation was induced. The result was consistent with C Leydolt’s study[10] which found IOP elevation caused a significant increase of axial eye length. At the same time, evidence has been found that near-work tasks can lead to transient axial elongation and subsequent transient myopia[11–14]. These phenomena aroused our interests of accommodation induced IOP changes and its influence on myopia progression in different populations. However, the sample size of the two studies mentioned above was relatively small and their subjects (progressing myopes and emmetropes in the first one[9] and only emmetropes in the second one[10]) were incomplete which impairs its conclusions to extend to different kinds of people.

The aim of this study is to investigate the *in vivo* IOP changes induced by 3 D accommodation in progressing myopes, stable myopes and emmetropes, to help us better interpret the role of accommodation and IOP variations in myopia development and progression.

## Methods

### Subjects

Through advertising recruitment, a total of 318 subjects participated in our study, among which 195 were progressive myopes (mean age 14.25 ± 6.86 years, mean spherical equivalent (SE) -4.06 ± 2.67D, range -12.50 D to -0.75 D), 75 were stable myopes (mean age 27.55 ± 9.17 years, mean SE -3.46 ± 1.60 D, range -8.00 D to -0.75 D) and 48 were emmetropes (mean age 27.63 ± 6.88 years, mean SE +0.01 ± 0.49 D, range -0.50 D to +0.50 D). Among the 318 subjects, 127 were males and 191 were females. Best corrected visual acuity (BCVA) of all the subjects was equal to or greater than 20/20 and no subjects exhibited astigmatism greater than 1.50 DC. The progressive myopic subjects were selected for myopia progression evidence of at least 0.50 D in the last 12 months prior to testing (based on present and previous refraction information), while the stable myopes were selected for myopia progression less than 0.50 D in the same period[15–18]. No subject reported any history of any ocular pathology, surgery, significant trauma or severe systematic diseases. None of the subjects had any medication that might have affected their accommodation or IOP. No subject reported any history of contact lenses wearing in the last 4 weeks prior to testing. Only the right eye of subjects was measured and analyzed.

### Ethics Statement

This study was approved by the Ethics Committee of Peking University Third Hospital. According to the tenets of the Declaration of Helsinki, verbal informed consent was obtained from all participants before entering the study. A specific investigator was designated to record the informed consent before subjects entering this study. And this informed consent procedure was approved by the Ethics Committee of Peking University Third Hospital.

### Procedure

Each subject underwent a general eye examination to ensure normal ocular health. Amplitude of accommodation was measured using the pushing-up test and subjects whose amplitude of accommodation were smaller than 6 D were excluded form this study. Then subjective cycloplegic refraction was carried out to determine their refractive status and best corrected visual acuity (BCVA).

IOP was measured using iCare rebound tonometry (iCare Company, Finland) under different accommodative statuses. The experiment protocol was described as following: First, subjects were fully corrected according to their subjective refraction outcomes. Then each subject wore an extra +3 D lens for 5 minutes to ensure subjects did not use any accommodation. IOP measurement was taken when the subject gazed at the first line test-object on the visual chart at 5-meter distance. Next each subject wore a lens of -3 D (on the fully corrected lenses) and gazed at the test-object for another 3 minutes to induce 3 D accommodation. The subject was told to try to see the test-object clearly and the timing was not started until the subject reported the target had become clear. Then we took off the right eye lens and measured the right eye IOP (with the left lens on). All the measurements were repeated for 3 times by the same researcher in the same examination room and the mean values of 3 times were recorded as the final IOP values. All the measurements were taken between 3 pm to 5 pm, with a sitting position.

### Statistical Analyses

All the values were recorded as mean ± standard deviation (SD) and approximately followed normal distribution. Correlation analysis was conducted to explore the interdependency of baseline IOP and age, gender and SE. One-way analysis of variance (ANOVA) and LSD test were employed to determine the difference of the baseline IOPs and IOP changes between progressing myopic, stable myopic and emmetropic groups. Since the age of subjects is significantly different (p<0.05) between groups, we conducted covariance analysis to exclude the age effect. Paired-t test was used to compare before and after IOPs when 3 D accommodation was induced within each group. Subgroup comparisons (among high, moderate and low myopes in progressing myopic group) were made using one-way ANOVA. Covariance analysis was also employed to exclude the effect of subjects’ age. Independent t-test was employed to determine the difference of IOP changes between adults and children in progressing myopic group. A significance level of α = 0.05 was employed in between and within group analyses. Considering multiple testing was present in subgroup analysis, Bonferroni correction was applied to adjust inflated Type I error rate[19], for which *p-values* <0.017 (i.e., 0.5/3 comparisons in 2 subgroups) and 0.008 (i.e., 0.5/6 comparisons in 3 subgroups) were deemed statistically significant[20].

## Results

The mean SE of progressing myopic group, stable myopic group and emmetropic group was -4.06 ± 2.67 D, -3.46 ±1.60 D and +0.01 ± 0.49 D (mean ± SD) respectively. The mean IOP change with accommodation for all subjects was -0.18 ± 2.13 mm Hg. Correlation analysis indicated there was a significant correlation between baseline IOP and subjects’ age (p = 0.046), but no significant correlations between baseline IOP and gender, baseline IOP and SE were detected (p = 0.962, p = 0.417). The age, gender and SE of each group and subgroup were presented in Tables 1 and 2.

**Table 1:** The demographic data of progressive myopes, stable myopes and emmetropes.

Group	N	Age (years)	Gender (male/female)	SE (D)
Progressive myopes	195	14.25±6.86	105/90	-4.06±2.67
Stable myopes	75	27.55±9.17	42/33	-3.46±1.60
Emmetropes	48	27.63±6.88	18/30	+0.01±0.49

This table showed the demographic data of progressing myopes, stable myopes and emmetropes.

**Table 2:** The demographic data of low, moderate and high progressing myopes.

Subgroup	N	Age (years)	Gender (male/female)	SE (D)
Low myopes	72	11.50±3.03	40/32	-1.73±0.65
Moderate myopes	77	11.84±2.65	37/40	-3.82±0.70
High myopes	46	22.57±9.15	28/18	-8.10±1.93
Children	153	11.35±2.18	79/74	-3.15±1.68
Adults	42	24.81±7.70	26/16	-7.38±2.98

This table showed the demographic data of low, moderate and high progressing myopes.

**Fig 1:**
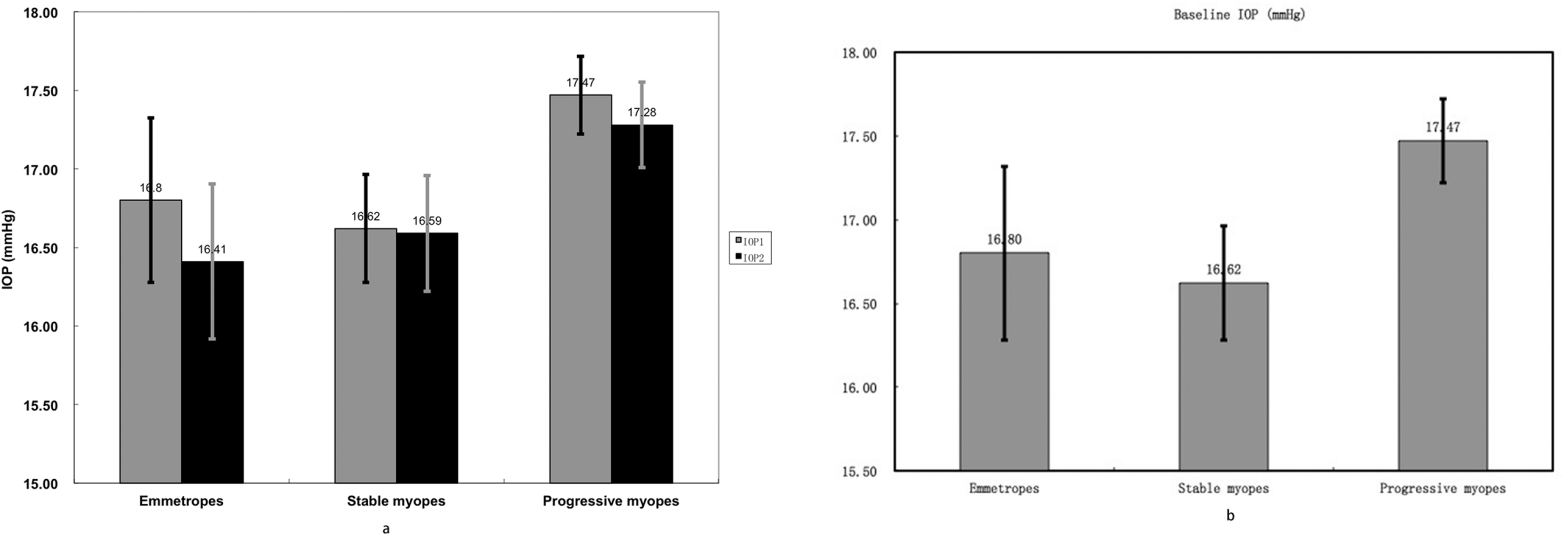
Fig 1a showed the IOP values before (IOP1) and after (IOP2) 3 D accommodation stimulus was presented in progressing myopic group, stable myopic group and emmetropic group. Fig 1b showed the accommodation induced IOP changes (IOPD) in 3 groups. IOP values decreased slightly when 3 D accommodation stimulus was presented, but the IOP changes were insignificant between groups (p>0.05). Error bars in the figs represent standard error of the data.

When at a relaxed accommodative status, mean IOP of progressing myopes was a little higher than stable myopes and emmetropes, but the difference was not significant (p = 0.133). After exclusion of age effect between groups, there was no significant difference between baseline IOPs (p = 0.183). When 3D accommodative stimulus was presented, the IOPs of the three groups all decreased a little, but still remained statistically insignificant (ANOVA and covariance analysis, p = 0.181, p = 0.582) (Table 3). The IOP changes between the three groups were not significant likewise (ANOVA and covariance analysis, p = 0.649, p = 0.415) (Fig 1).

**Table 3:** The IOP of three groups before and after accommodation.

Group	Accommodation relaxed (mmHg)	3 D accommodative stimulus (mmHg)	IOP changes (mmHg)
Progressive myopes	17.47±3.46	17.28±3.79	-0.19±2.16
Stable myopes	16.62±2.98	16.59±3.19	-0.03±1.68
Emmetropes	16.80±3.62	16.41±3.42	-0.39±2.65
P (one-way ANOVA)	0.13	0.18	0.65
P (covariance analysis)	0.18	0.58	0.42

The IOP of progressive myopes, stable myopes and emmetropes before and after 3 D accommodative stimulus were presented. One-way ANOVA and covariance analysis were employed to determine the between-group difference of IOPs. No statistically significant difference was detected between groups.

**Fig 2:**
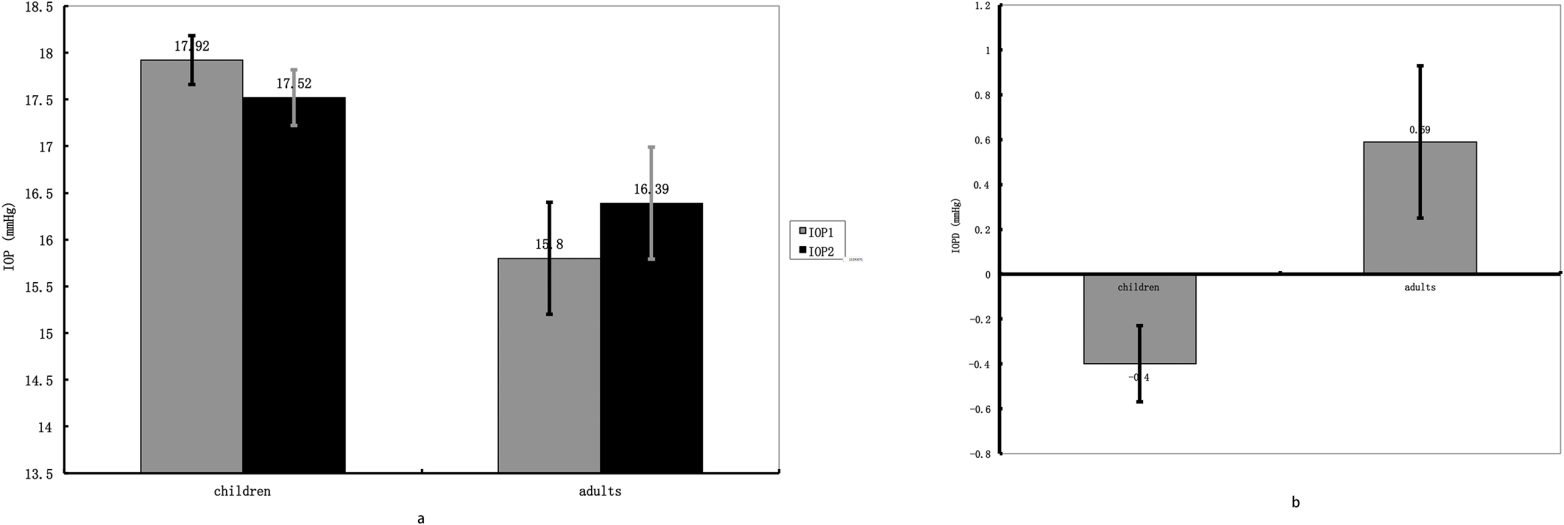
Fig 2a showed the IOP values before (IOP1) and after (IOP2) 3 D accommodation stimulus was presented in subgroups (children progressing myopic group vs. adults progressing myopic group). Fig 2b showed the IOP changes (IOPD) in the 2 subgroups. IOP decreased in children progressing myopic group but increased in adults progressing myopic group after 3 D accommodation was induced. The difference between IOP changes was significant between the 2 groups (p = 0.008). Error bars in the figs represent standard error of the data.

**Fig 3:**
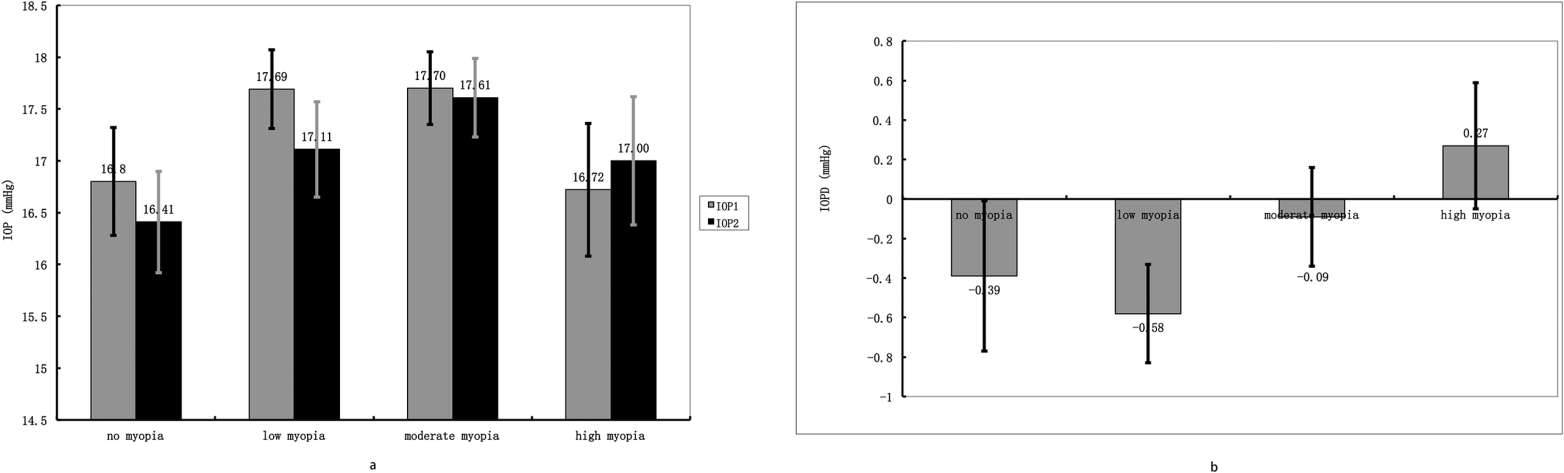
Fig 3a showed the IOP values before (IOP1) and after (IOP2) 3 D accommodation stimulus was presented in subgroups (low progressing myopic group, moderate progressing myopic group, high progressing myopic group and emmetropic group). Fig 3b showed the IOP changes (IOPD) in the 2 subgroups. IOP decreased in low and moderate progressing myopic group, as in emmetropic group, but increased in high progressing myopic group after 3 D accommodation was induced. The difference between IOP changes was insignificant between the 4 groups (p = 0.838). Error bars in the figs represent standard error of the data.

Subgroup analysis showed that children (<18 years old) and adults (≥18 years old) acted differently in progressing myopic group. IOP values of children and adults before and after accommodation were presented in Table 4. IOP of children in this group acted similarly like emmetropes and stable myopes–IOP decreased slightly (-0.41 ± 2.10 mmHg) with accommodation. Unlike subjects mentioned above, IOP of adults in progressing myopic group increased by 0.59 ± 2.17 mmHg after 3 D accommodation was induced for 3 minutes. Independent Student t-test indicated that the IOP changes between children and adults were highly significant (p = 0.008). Fig 2 showed the IOPs and IOP changes in children and adults subgroups. We also compared the IOP changes between adults with progressing myopia and adults with stable myopia. However, no significant difference was found (p = 0.116). Another subgroup analysis divided progressing myopic group into high myopes (SE ≤ -6 D), moderate myopes (-6 D < SE ≤ -3 D) and low myopes (-3 D <SE < -0.5 D) and compared their IOP values under each condition. Table 5 and Fig 3 displayed IOPs before and after accommodation, as well as IOP changes in each group. One-way ANOVA showed no statistical significance was detected in IOP changes between the 3 subgroups and the emmetropic group (p = 0.095). Similar result was produced in covariance analysis (p = 0.838).

**Table 4:** The IOP of children and adults subgroups before and after accommodation.

Subgroup	Accommodation relaxed (mmHg)	3 D accommodative stimulus (mmHg)	IOP changes (mmHg)
Children	17.92±3.20	17.52±3.74	-0.40±2.11
Adults	15.80±3.89	16.39±3.88	+0.59±2.17
P (independent t test)	0.00	0.09	0.01

The IOP of children and adults progressing myopes before and after 3 D accommodative stimulus were presented. Independent t test were employed to determine the IOP difference of the 2 subgroups. We find there is a statistically significant difference between children and adults progressing myopes in IOP changes (p = 0.01).

**Table 5:** The IOP of three subgroups before and after accommodation.

Subgroup	Accommodation relaxed (mmHg)	3 D accommodative stimulus (mmHg)	IOP changes (mmHg)
Low myopes	17.69±3.18	17.11±3.93	-0.58±2.08
Moderate myopes	17.70±3.10	17.61±3.37	-0.09±2.18
High myopes	16.72±4.32	17.00±4.24	+0.27±2.17
P (one-way ANOVA)	0.25	0.61	0.10
P (covariance analysis)	0.07	0.20	0.84

The IOP of low, moderate and high progressing myopes before and after 3 D accommodative stimulus were presented. One-way ANOVA and covariance analysis were employed to determine the between-subgroup difference of IOPs. No statistically significant difference was detected between subgroups.

## Discussion

Our study shows that no significant difference of IOPs is detected without any accommodation or with 3 D accommodation between progressing myopic group, stable myopic group and emmetropic group. Correlation analysis also shows SE is not significantly correlated to baseline IOPs. This result is consistent with several previous studies[5, 7, 21], which questioned the role of elevated baseline IOP levels in the development of axial elongation and myopia. No positive association between IOP and AL indicated that myopia pathogenesis might act independently of baseline IOP in the general population.

Moreover, IOP decreased a little (though insignificantly) in all three groups when 3 D accommodation was induced for 3 minutes. And the IOP changes between groups were also insignificant, which is different from our previous report^9^ in which IOP of young progressing myopes rose with accommodation, while that of emmetropes dropped a little. However, this result is to some extent consistent with several other studies[22, 23]. Read SA[23] found IOP reduced significantly as a result of the accommodation task when utilised the DCT instrument. In a tonographic study, Armaly[22] found significant changes in aqueous humour dynamics occur as a result of accommodation, with increases in both aqueous outflow and inflow facility. In that study, aqueous outflow was in a dominant position so that the overall IOP decreased with accommodation. In general, accommodation induced IOP decrease could be attributed to the mechanical effect of ciliary muscle contraction upon the outflow apparatus. But the amplitude of IOP changes in our study (only -0.18 ± 2.13 mm Hg) is much smaller than others, with little clinical significance. In this study, we find that accommodation induced IOP variation is negligible in most cases.

Subgroup analysis shows IOP acted differently between children and adults progressing myopes. IOP of children, as well as of low, moderate progressing myopes and emmetropes declined with accommodation while IOP of adult and high progressing myopes ascended. We notice that previous studies mainly focused on young mild and moderate myopes and emmetropes, with few data of persistently progressing high myopia in adults. To our knowledge, this is the first study that reports the divergence of IOP reaction in different populations. The reasons of divergent IOP reaction are complicated. One reason might be related to structural and functional differences of ciliary muscle, trabecular meshwork and anterior chamber angle between children and adults myopes. Besides, our subjects are Chinese and most subjects in previous studies were non-Chinese. To our general knowledge, Chinese are more susceptible to myopia than other races[24]. This discrepant IOP reaction to accommodation might be due to anatomic and racial differences. It was well accepted that myopia progression tended to stop after one headed into adulthood[25]. Our study indicates that accommodation induced IOP elevation in adult population might be partly responsible for their persistent myopia progressing which often resulted in a relatively high refractive error and long axial length. This process might be mediated by mechanical force of elevated IOP and subsequent axial length (AL) elongation, which is consistent with Read SA and Chakraborty R’s report that AL would change with IOP variations[26, 27].

This study has included a relatively large sample of subjects over a broad age and RE range and obtained reproducible refractive and IOP measures. However, it is not spared from some methodological limitations. Firstly, although IOP measurements were repeated for 3 times by the same experienced operator, we could not exclude the impact of repeatability limits of ICare rebound tonometer on our results. Many previous studies[28–30] reported a good consistency of IOP measurements between ICare tomometer and Goldmann tomometer. But it was reported[31] the measuring position (central or peripheral) and angle (straight or angled) might have an influence on the measurements. Apart from that, to our knowledge, IOP measurement is evidently influenced by corneal thickness (CT). But we fail to compare the corneal thickness between groups to exclude its effect on IOP measurement. Secondly, correlation analysis indicates that IOP value is correlated with age factor. But there is significant difference of age between progressing myopes, stable myopes and emmetropes. And we encountered the similar condition when subgroup analysis is made (between high, moderate and low progressing myopes). To exclude the age effect, we employ covariance analysis in IOP comparisons[32] and explain the results with caution. Moreover, because of the nature of our cross-sectional study, it is impossible for us to determine the causal relationship between accommodation induced IOP variation and myopia progression, especially in adult high progressing myopes. Finally, we notice that even in progressing myopic group, the IOP changes of adults is within 1 mmHg (+0.59±2.17 mmHg). The clinical meaning of this minor IOP change is unclear. In other word, based on our present results, we are not certain about its influence on persistent myopia progression, which can only be confirmed in future longitudinal studies.

In summary, this study investigates the IOP variations induced by 3 D accommodation in different populations. We find the IOP experienced a transient descending in low and moderate myopes, child progressing myopes and emmetropes, but increased in adult progressing myopes when accommodation was induced. Our study indicates that IOP elevation after accommodation might be related to persistent progression in adult myopes in a biomechanical way. But the exact mechanism of myopia progression is not clarified. More detailed longitudinal studies were needed to confirm this issue.

## Supporting Information

S1 Appendix Demographic information and data of the whole participants. All relevant data could be found within the paper and its Supporting Information file Database.
(ZIP)
